# Supplementary material for: Characterization of Cancer Stem Cells in Laryngeal Squamous Cell Carcinoma by Single-cell RNA Sequencing
Source: Genomics Proteomics Bioinformatics. 2024 Aug 6;22(4):qzae056. doi: 10.1093/gpbjnl/qzae056 (PMC11522873; doi:10.1093/gpbjnl/qzae056)
Supplement: qzae056_Supplementary_Data [file qzae056_supplementary_data.zip › supplementary material captions.docx]

**Supplementary material**

**Figure S1 scRNA-seq analysis in LSCC samples**

**A.** Point plot shows the number of genes and UMIs in each cell. Each dot represents a single cell; the color of each dot is according to the percentage of mitochondrial genes in the cell. **B.** The absolute number of cells from each patient, including doublets, high-quality cells, and low-quality cells. The high-quality cells were selected for downstream analysis. **C.** The *t*-SNE plot shows the authority marker genes expression in eight major cell types. **D.** Average proportions of different cell types in LC, LM, and PT. **E.** GO enrichment analysis based on the downregulated genes of epithelial cells in LC compared to those in PT (adjusted *P* value < 0.01, log_2_(FC) > 1). UMIs, unique molecular identifiers;

**Figure S2 Characteristics of the epithelial cell subpopulations and CSC in LSCC**

**A.** Dot plot shows the expression of marker genes in all the epithelial cell subpopulations. **B.** Bar plot shows the absolute number of all the epithelial cell subpopulations in different cell cycle phases. **C.** GSVA scores of EMT gene sets in the epithelial cell subpopulations in LSCC. **D.** The enriched GO terms for marker genes in SC-C2. **E.** The enriched GO terms for marker genes in SC-C1. **F.** Differential expression analysis between LC and PT tissues in CSCs. **G**. The distribution of CSC-like cells and NSC-like cells in different tissue type from an independent scRNA-seq dataset (GES206332). EMT, epithelial–mesenchymal transition.

**Figure S3 CSC marker gene signatures and clinical application in LSCC**

**A.** Function enrichment analysis results for the CSC marker genes. **B.** The values of lambda used in the 10-fold cross-validation for glmnet. **C.** The ROC curves show predicted 1-, 3-, and 5-year overall survival of the LSCC patients based on prognostic model in the training and testing datasets.

**Figure S4 Analysis of the stroma cell subpopulations and The cell–cell communication between stroma cells and CSCs in LSCC**

**A.** Violin plot showed the conventional marker genes and differentially expressed genes in different subpopulations of T cells. **B.** Heat map showed the differentially expressed genes in different subpopulation of myeloid cells. **C.** Violin plot shows the conventional marker genes and differentially expressed genes in different subpopulation of B cells. **D.** Dot plot shows differentially expressed genes in different subpopulations of endothelial cells. **E.** Heat map shows differentially expressed genes in different subpopulations of fibroblast cells. **F.** Volcano plot shows differentially expressed genes between *SDC4*^high^ and *SDC4*^low^ groups of CSCs.

**Table S1 The clinical information and pathologic features of patients**

**Table S2 The differentially expressed genes of epithelial cells between LC and PT**

**Table S3 The differentially expressed genes of SC-C1 and SC-C2**

**Table S4 Cancer stem cell gene signatures**

**Table S5 Top 50 Cancer Therapeutics Response Signatures that connected with the CSC-specifically expressed genes**
