# Supplementary figures and images for: Characterization of Cancer Stem Cells in Laryngeal Squamous Cell Carcinoma by Single-cell RNA Sequencing
Source: Genomics Proteomics Bioinformatics. 2024 Aug 6;22(4):qzae056. doi: 10.1093/gpbjnl/qzae056 (PMC11522873; doi:10.1093/gpbjnl/qzae056)

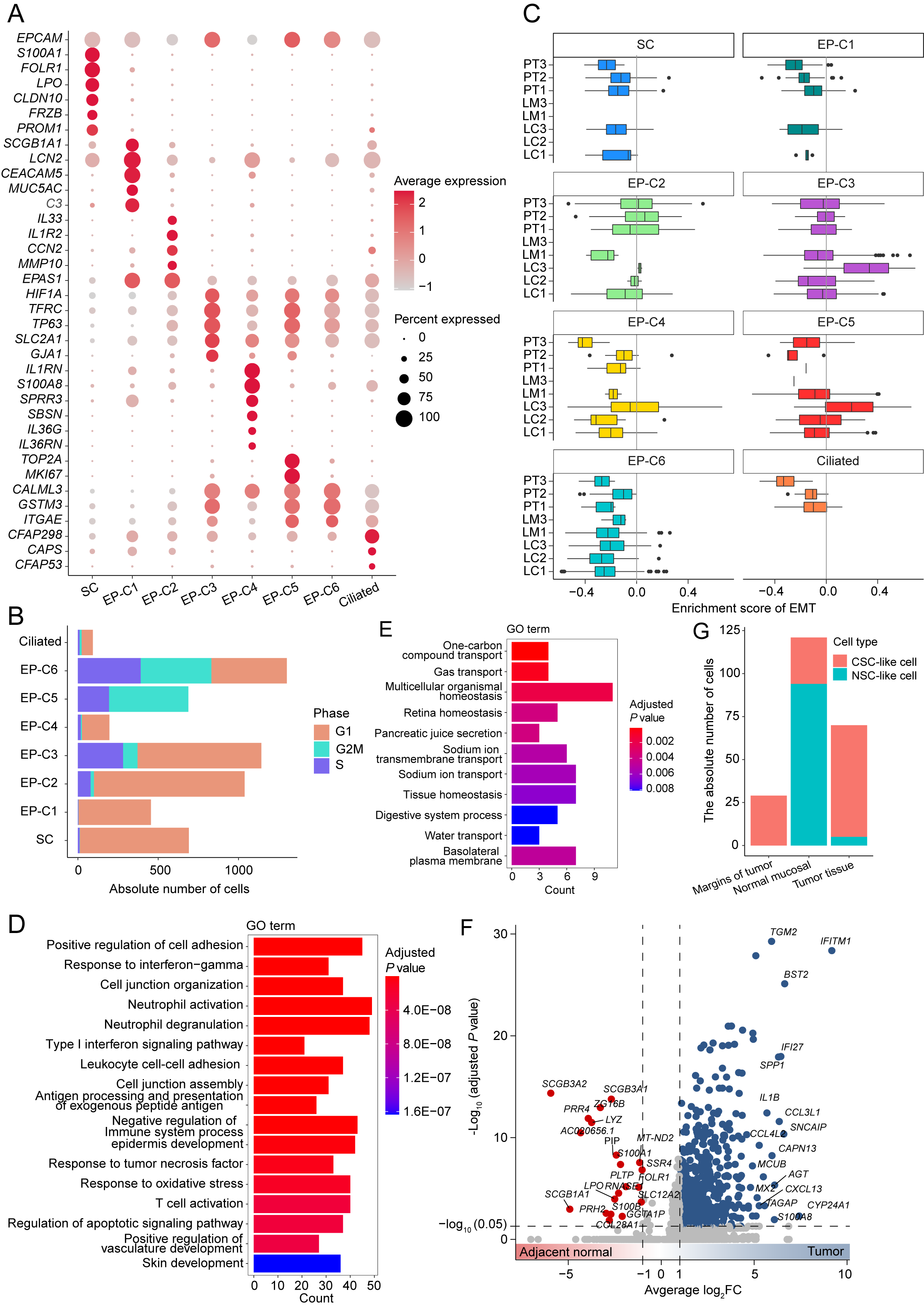

Supplement: qzae056_Supplementary_Data [file qzae056_supplementary_data.zip › Figure S2.tif]

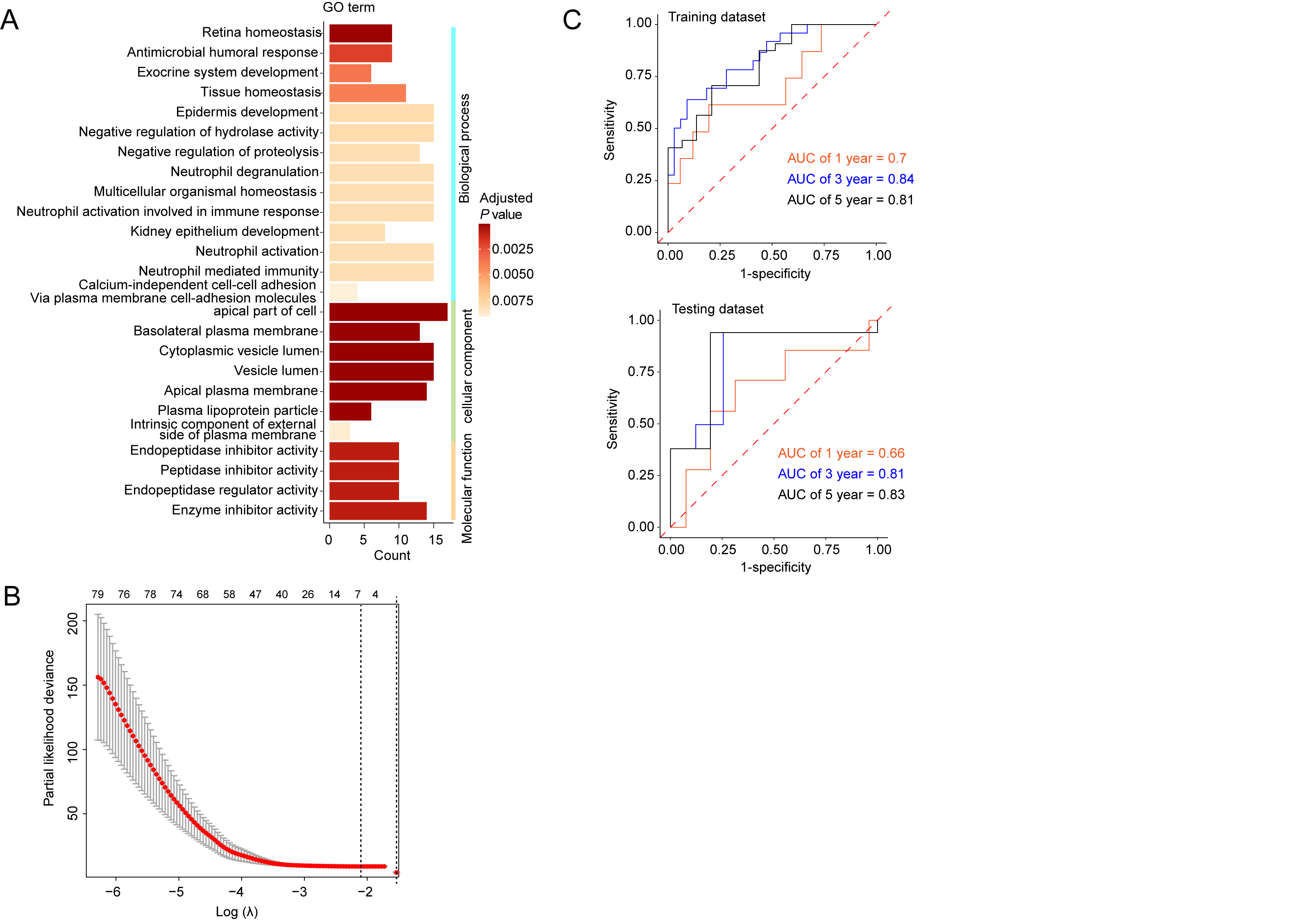

Supplement: qzae056_Supplementary_Data [file qzae056_supplementary_data.zip › Figure S3.tif]

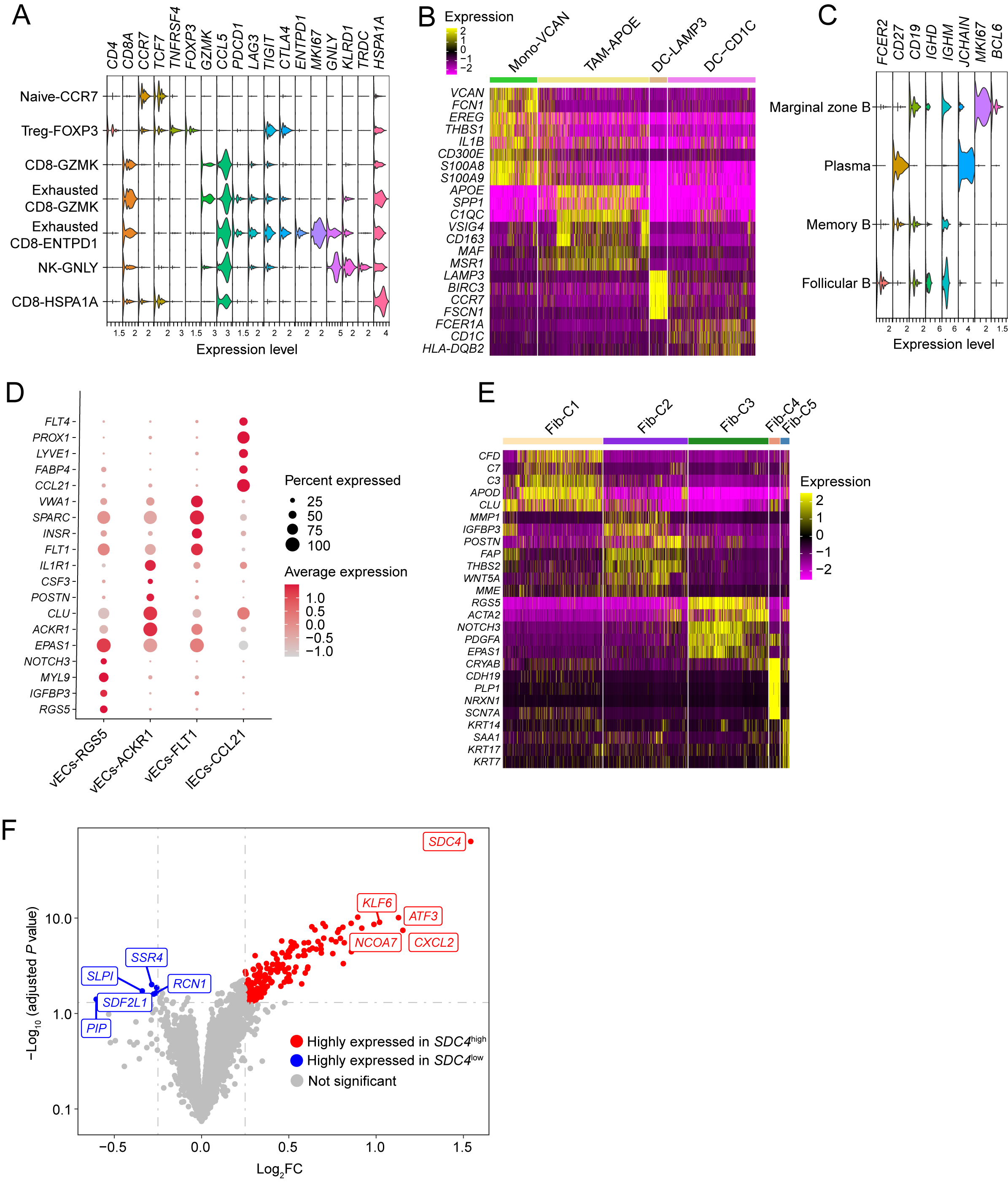

Supplement: qzae056_Supplementary_Data [file qzae056_supplementary_data.zip › Figure S4.tif]

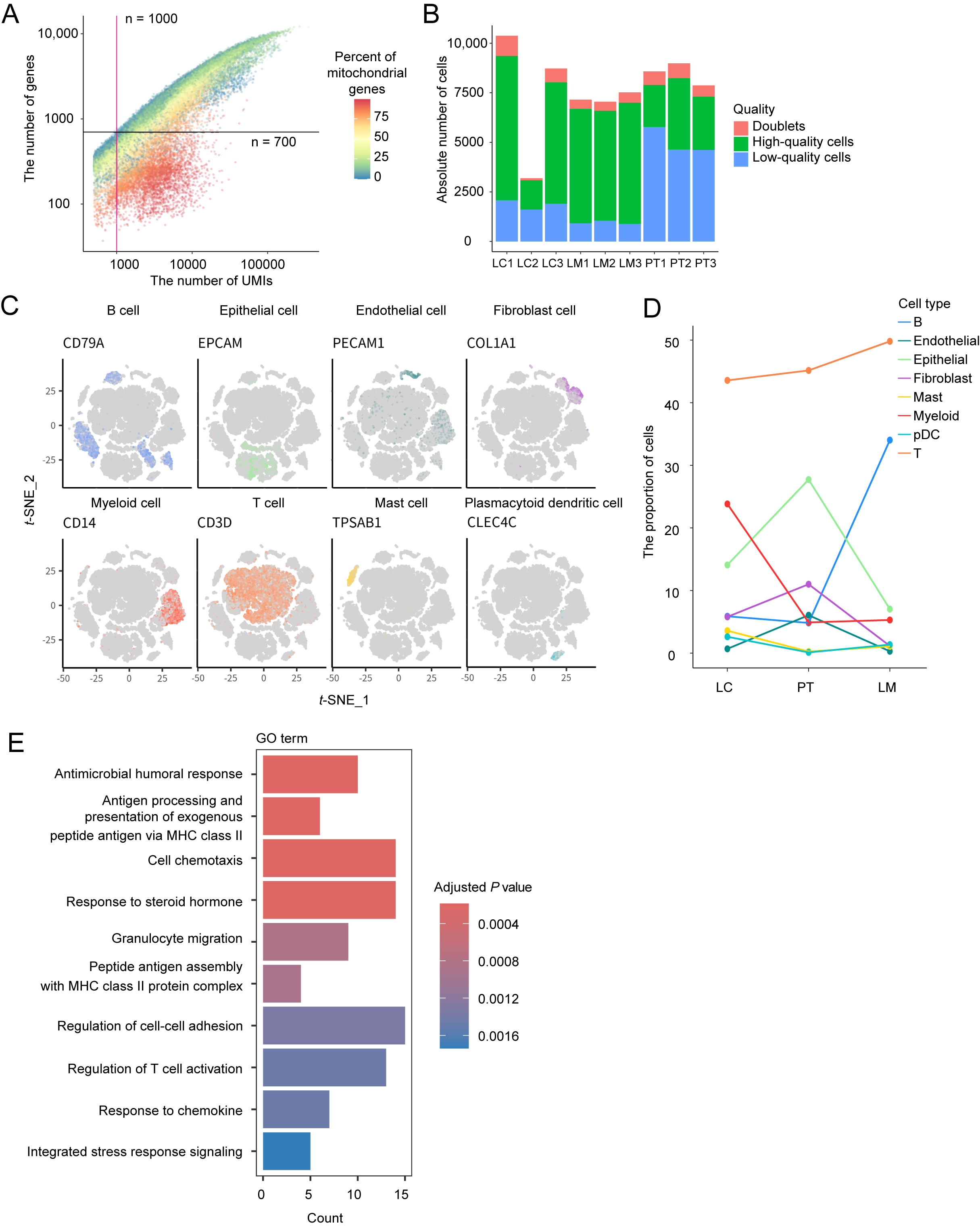

Supplement: qzae056_Supplementary_Data [file qzae056_supplementary_data.zip › Figure S1.tif]
